# Influencing factors and mediating mechanisms of job crafting in clinical nursing practice

**DOI:** 10.3389/fpubh.2025.1711339

**Published:** 2025-12-18

**Authors:** Jia Wang, Yaru Li, Jiaxin Sun, Yajuan Cui, Mei Su, Peijuan Tang, Yanting Wang, Yuchong Hu, Wenzhong Chang, Yanli Wang

**Affiliations:** 1 Department of Gynaecology, Inner Mongolia People’s Hospital, Hohhot, Inner Mongolia, China; 2 Department of Nursing, Ordos Traditional Chinese Medicine Hospital, Ordos City, Inner Mongolia, China; 3 Department of Clinical Medical Research Center, Affiliated Hospital of Inner Mongolia Medical University, Hohhot, Inner Mongolia, China; 4 School of Nursing, Inner Mongolia Medical University, Hohhot, Inner Mongolia, China; 5 Department of Nursing, Inner Mongolia People’s Hospital, Hohhot, Inner Mongolia, China

**Keywords:** nurses, job crafting, career calling, TIPI-C, mediation

## Abstract

**Aim:**

To investigate the factors influencing job crafting levels among clinical nurses and elucidate the internal mechanisms linking personality traits to job crafting.

**Background:**

Job crafting plays a pivotal role in nursing management, with its optimization having the potential to enhance the quality of clinical care, the efficacy of healthcare organizations, and the multifaceted professional development of nurses.

**Design:**

A cross-sectional design.

**Methods:**

This study utilized a sample of 1,273 clinical nurses from tertiary hospitals in Inner Mongolia, China, selected through multistage sampling. To analyze the influencing factors and pathways, multiple linear regression and the Hayes PROCESS macro program (Models 4) were employed.

**Results:**

The study reveals that clinical nurses engage in job crafting to a moderate to high degree, with significant influences from career calling, extraversion, conscientiousness, and nurse regulatory focus. Furthermore, the mediating role of regulatory focus in the relationship between extraversion/conscientiousness and job crafting highlights the distinct mechanisms through which these personality traits shape occupational behaviors.

**Conclusion:**

The study identifies multiple factors affecting nurses’ job crafting. It is imperative for nursing managers to comprehensively consider these factors and develop a targeted monitoring system to effectively enhance nurses’ capacity for job crafting.

**Relevance to clinical practice:**

The findings of this study offer theoretical support for the optimization of nurses’ job crafting strategies and may also draw increased attention to the mental health and sustainable career development of the nursing workforce.

**Patient or public contribution:**

No patient or public contribution.

## 1 Introduction

Job crafting refers to the proactive behavior in which individuals take the initiative to modify the nature and scope of their work. It is recognized as a positive factor in both academic research and social contexts. Studies show that job crafting can enhance work engagement (1), stimulate innovative behaviors (2), and increase organizational commitment (3). The International Labour Organization’s Decent Work Country Programme for China (2023–2025) emphasizes proactive job crafting as a key strategy for promoting employee empowerment and establishing a sustainable work system (4). However, job crafting presents challenges within the nursing profession. Nurses’ ability to reshape their careers is often constrained by prolonged exposure to high-stress environments, including illness, disability, and death. Research indicates that nurses who lack the capacity to engage in job crafting experience a 41% higher incidence of burnout compared to those who actively reshape their roles. Furthermore, they report significantly lower job satisfaction (5). This lack of job crafting competence leads to rigidity in nursing practices, impedes clinical innovation, and results in role maladjustment, perpetuating a harmful cycle known as the “Nursing Efficacy Deficit.” The World Health Organization (WHO) has identified an escalating crisis in nursing human resources, forecasting a global shortage of over 7.6 million nurses by 2030 (6). This underscores the urgent need to strengthen nurses’ ability to reshape their work roles to address the growing challenges in the healthcare sector.

To optimize the management of nursing human resources and enhance professional efficacy, it is crucial to identify the factors influencing nurses’ job crafting behaviors. Numerous studies have highlighted various factors that affect job crafting among nurses. For example, research conducted on ICU nurses in Beijing found that those with a bachelor’s degree or higher were more likely to enhance their sense of value by restructuring their work tasks and cognitive frameworks (7). In contrast, nurses in poorer health tended to reduce their workloads to maintain psychological balance (8). Additionally, family structure played a significant role, with nurses who had young children often adjusting their shift schedules to alleviate work–family conflicts (9). While existing studies have clarified the impact of explicit factors such as education and health status on nurses’ job crafting, there remains a gap in understanding the underlying psychological mechanisms that drive these behaviors.

Grounded in the Job Demands-Resources (JD-R) model, an occupational stress and well - being framework that posits two core categories of job characteristics (job demands and job resources), this study introduces two key variables: the Big Five personality traits and regulatory focus. It seeks to examine their mediating roles within the JD-R framework, focusing on individual differences and cognitive regulation. The Big Five personality traits, as stable psychological dispositions, offer insight into the substantial variations in nurses’ behaviors, including task crafting and relational crafting. Regulatory focus, on the other hand, explains individuals’ differential responses to job resources and demands through two cognitive tendencies: approaching success (promotion focus) and avoiding failure (prevention focus) (10). This study aims to identify the factors influencing clinical nurses’ job crafting and, based on the JD-R theory, explores the impact of the Big Five personality traits on job crafting. It further examines the mediating role of regulatory focus in this relationship, providing valuable intervention targets for nursing human resource management to enhance care quality.

## 2 Background

The concept of “job crafting” was first introduced by Dutton (3), emphasizing the dynamic process of individual value creation through task, relationship, and cognitive crafting. Empirical research has shown a strong association between job crafting and nursing groups, with such proactive job adjustments being common among nurses (8). Effective job crafting not only helps mitigate burnout but also strengthens nurses’ sense of belonging to the hospital. This, in turn, leads to two key benefits: improved quality of nursing services and enhanced patient recovery (9, 11). As a result, job crafting has been described as the “stabilizer of nursing quality.”

Job crafting has emerged as a key strategy for optimizing nursing team effectiveness. Studies show that this proactive behavior, which involves flexible adjustments to work content, interpersonal relationships, and cognitive perspectives, can significantly enhance nurses’ performance and stress resilience (12). Evidence from various clinical settings highlights the benefits of job crafting. For example, emergency department nurses reduced burnout risk by reorganizing task divisions (13), while young nurses in rural U. S. hospitals increased work engagement by altering their perceptions of stress during the epidemic (14). Additionally, strategically redistributing tasks and optimizing workflow allow nurses to manage their workloads more effectively, leading to reduced burnout and higher job satisfaction (15). In contrast, a lack of ability to reorganize work can negatively impact the quality of care, reduce job satisfaction, and increase turnover rates among nurses (11, 16).

Numerous factors influence job crafting among nurses. The JD-R theory, a key framework for understanding occupational stress and employee well-being, emphasizes the dynamic balance between job demands and resources in shaping individual behavior (17). According to this theory, job demands deplete psychological resources, while job resources help alleviate stress and promote positive psychological states. In 2017, the JD-R theory was applied to study job crafting within the nursing profession (18). The findings revealed that when nurses proactively adjusted their job content through strategies such as task expansion and skill optimization, their control over resources increased, which in turn reduced the risk of burnout caused by high job demands. Additionally, nurses working in healthcare institutions with strong organizational support systems were better able to convert work resources into career satisfaction and were less likely to leave due to resource depletion. This was achieved by engaging in knowledge sharing and teamwork (19).

According to the JD-R theory, personality traits represent an important personal resource and a key factor influencing job crafting. These traits are commonly conceptualized within five core dimensions in psychology—extraversion, conscientiousness, openness, emotional stability, and agreeableness—which capture individual differences in personality. Personality traits affect nurses’ professional adjustment, job performance, and the quality of patient care by shaping attributes such as emotional regulation, social interaction, and responsibility. Research has shown that extraversion facilitates role reconfiguration by expanding social networks (20); conscientiousness enables nurses to maintain effective performance in complex tasks through goal-directed self-regulation (21); openness promotes creative adaptation and supports the development of individualized care plans (20); and emotional stability buffers the negative effects of workplace uncertainty and stress (22). These findings highlight the distinct influence of different personality traits on nurses’ job crafting behaviors. Furthermore, personality traits are closely linked to an individual’s sense of career calling by shaping the perceived meaning of their work. This sense of vocational significance reinforces professional commitment and adaptability, creating a positive cycle of personality-driven motivation (23).

The concept of career calling, defined as an individual’s profound and meaningful connection to the nursing profession, serves as a continuous source of motivation for job crafting. It encourages the internalization of altruistic values and fosters a strong sense of mission (10). Unlike stable personality traits, career calling demonstrates significant dynamic adaptability. Nurses with a strong sense of career calling are able to transform standardized procedures into opportunities for patient empowerment, build therapeutic alliances in nurse–patient interactions, and reframe burdensome tasks as acts of life-care (24, 25). This meaning-driven reframing produces a dual buffering effect, reducing the impact of emotional exhaustion on work engagement and overcoming institutional constraints by redefining psychological contracts. In the collectivist context of China, the influence of career calling is further amplified by cultural factors. The Confucian ethic of benevolence and love aligns with the organizational value of dedication, raising nurses’ professional mission to include upholding family honor and fulfilling social roles (26).

According to the JD-R theory, the interaction between an individual’s career calling and available job resources drives the development of goal-oriented strategies aimed at achieving career value identity. Regulatory focus, a key framework for goal setting, reflects the fundamental motivations of striving for growth or avoiding risks in career pursuits (27). The process of behavioral decision-making in response to a career calling, which involves seeking meaning, differs according to the individual’s regulatory focus. Empirical evidence shows that nurses with a promotion focus are better at converting career-related pressures into growth opportunities, exhibiting significantly higher levels of career satisfaction and innovative behaviors compared to those with a prevention focus (28, 29). Promotion focus is positively correlated with job crafting behaviors, while prevention focus is associated with more adaptive crafting (30). Additionally, longitudinal research tracking a two-year follow-up suggests that a prevention focus weakens the positive effects of job crafting on career growth, while a promotion focus strengthens this relationship (31). Thus, regulatory focus plays a critical moderating role in the context of job crafting.

In conclusion, job crafting among nurses is influenced by a variety of factors. However, existing research largely examines the independent effects of individual factors and lacks a comprehensive analysis of the synergistic interactions between these factors. To address this gap, the present study aims to answer three core questions from an integrative perspective: (1) What are the characteristics of job crafting within the nursing population, and are there group-based differences? (2) What factors influence nurses’ job crafting? (3) Are there interactions among these influencing factors? This study proposes a three-dimensional analytical framework (status quo, motivators, mechanisms) to overcome the limitations of single-factor research. By doing so, it seeks to provide a scientific basis for nursing managers to develop systematic intervention strategies and contribute to advancing the theoretical understanding of job crafting within healthcare settings.

## 3 Methods

### 3.1 Aims

To investigate the factors influencing job crafting levels among clinical nurses and elucidate the internal mechanisms linking personality traits to job crafting.

### 3.2 Study design and participants

A multicenter cross-sectional study was conducted from February to March 2025 in Inner Mongolia, China. The sample size was determined using the standard formula for cross-sectional studies: *n* = *Z*^2^ × *p* × (1 − *p*)/*e*^2^, where *Z* represents the Z-score corresponding to the desired confidence level (commonly 1.96 for a 95% confidence interval), *p* denotes the anticipated proportion (set at 0.5 to ensure the maximum sample size), and *e* signifies the allowable margin of error (typically 5%, or 0.05). Substituting these values into the formula yields an initial sample size of approximately 384. To account for the design effect associated with cluster sampling, the sample size is typically increased by a factor of 1.5 to 2, resulting in an adjusted sample size range of 576 to 768.

This study employed a three-stage stratified sampling method. Initially, six league cities/prefecture-level cities were stratified based on the regional divisions of the Inner Mongolia into eastern, central, and western regions. Subsequently, 12 tertiary-level hospitals were stratified according to the distribution of regional tertiary-level hospitals. Finally, within the selected hospitals, nurses were chosen using a cluster sampling method, stratified by hospital size (number of beds) and department type. A detailed description of the sampling method is provided in Supplementary file S1. Ultimately, 1,273 nurses were selected. The inclusion criteria were: (1) registered nurses with a valid nursing practice certificate; (2) a minimum of one year of clinical work experience; and (3) informed consent and voluntary participation. The exclusion criteria were: (1) registered nurses undergoing training, further education, or internships; (2) absence from work during the survey period due to reasons such as vacation, study leave, illness, or childbirth; and (3) inability to complete the survey, such as experiencing a major life event. This cross-sectional study was conducted in accordance with the STROBE guidelines. The completed checklist is provided as supplementary material (STROBE-checklist-v4-cross-sectional.pdf).

### 3.3 Data collection

This study utilized the WJX platform (Questionnaire Star^1^) for data collection, implementing stringent quality control measures to ensure the validity and reliability of the data. During the questionnaire design phase, the initial version was developed and refined through a comprehensive literature review and consultations with experts. In the distribution and collection phase, several measures were applied: submissions were restricted to one per IP address, WeChat ID binding was enabled to prevent duplicate responses, and a minimum submission time of 90 s was set to intercept rapid, non-reflective responses. During the data cleaning phase, invalid responses—such as straightlining or patterned responses (*n* = 60), logical inconsistencies (*n* = 40), and failed attention checks (*n* = 73)—were excluded. Out of a total of 1,446 collected questionnaires, 1,273 valid responses were retained after this screening process, resulting in an effective retrieval rate of 88.04%.

### 3.4 Instruments

#### 3.4.1 Demographic and sociological factors

Following an extensive review of the literature, the research team developed and administered a comprehensive general information questionnaire. This questionnaire encompassed various variables, including department type, ethnicity, age, gender, education level, marital status, professional title, job position, night shifts per month, total years of work experience, employment status, self-perceived health status, and frequency of off-site training per year.

#### 3.4.2 Job crafting scale

This scale is a multidimensional assessment tool originally developed by Dvorak’s team (32) and subsequently adapted for cross-cultural contexts by Zhu Shixiao’s team (33). It comprises three sub-dimensions: relational crafting, task crafting, and cognitive crafting, with a total of 21 items. Responses are measured on a 5-point Likert scale (1 = strongly disagree, 5 = strongly agree), with the aggregate score indicating the individual’s level of job crafting. The original scale demonstrated an internal consistency coefficient of 0.91, which was enhanced to 0.98 in the present study, with a Guttman split-half coefficient of 0.92.

#### 3.4.3 The career calling scale

This scale derived from Dobrow’s theoretical framework (34), has been adapted into Chinese by Pei Yujing (35). This 12-item instrument employs a 5-point Likert scale, featuring representative items such as “I am willing to make sacrifices for my current job.” The original scale demonstrated a reliability coefficient of 0.94, while the current study reported a Cronbach’s α of 0.98 and a Guttman split-half coefficient of 0.96, indicating a high level of reliability.

#### 3.4.4 Nurse regulatory focus scale

Nurse regulatory focus scale (NRFQ) was originally developed by Wallace and Chen (36) and subsequently localized and validated by Li Shuying’s team (37). This scale is structured into two dimensions: promotion focus and prevention focus. It comprises 12 items, each rated on a 5-point Likert scale, yielding a total score range of 12 to 60. In the present study, the scale demonstrated high internal consistency, with a Cronbach’s alpha of 0.97 and a Guttman split-half coefficient of 0.95. These metrics indicate the scale’s efficacy in distinguishing the behavioral characteristics of caregivers with varying regulatory focus orientations.

#### 3.4.5 The psychological capital questionnaire

The Chinese version of Luthans’ classic scale (38) was validated for applicability to nursing groups by Luo Hong’s team (39). This instrument encompasses 20 items across four dimensions: self-efficacy, hope, resilience, and optimism, and is rated on a 6-point scale. The overall reliability of the scale was 0.92, with subscale alpha coefficients ranging from 0.718 to 0.890, and a test–retest reliability of 0.82. In this study, the Cronbach’s alpha was found to be 0.99 and a Guttman split-half coefficient of 0.98, indicating excellent reliability.

#### 3.4.6 Ten-item personality inventory in China

This scale was developed following the methodology established by Gosling et al. (40). This inventory, validated by Jinde (41), utilizes a 10-item condensed version of the personality assessment tool to evaluate dimensions such as extraversion, agreeableness, conscientiousness, emotional stability, and openness, using a 7-point scale (1 = absolute disagreement, 7 = absolute agreement). Items 1, 3, 5, 7, and 9 are scored positively, while the remaining items are scored negatively. Each personality dimension is represented by a specific combination of items; for example, extraversion is assessed using items 1 and 6. The Cronbach’s *α* for this inventory was measured as 0.86 and a Guttman split-half coefficient of 0.88. It is important to note that for a brief measure such as the TIPI, assessing reliability at the dimension level provides more meaningful insights. Therefore, we computed the inter-item correlations for each two-item dimension. The results revealed statistically significant correlations for all dimensions (*p* < 0.01), specifically: Extraversion (*r* = 0.15), Agreeableness (*r* = 0.20), Conscientiousness (*r* = 0.07), Emotional Stability (*r* = 0.08), and Openness (*r* = 0.18). Although these correlations were significant, their magnitudes were generally low (all below 0.40). This is consistent with the known characteristics of the TIPI (42), which represents a trade-off between assessment efficiency and the internal consistency typically achieved by traditional multi-item scales. Consequently, it is important to recognize that the measurement of personality traits in this study contains some degree of error, a limitation that should be considered when interpreting findings related to these constructs.

### 3.5 Data analysis

Data management was performed using Microsoft Excel 2019, and statistical analyses were conducted with SPSS Statistics 24.0 and Mplus 8.3. Categorical variables were described using frequencies (*n*) and percentages (%), while continuous variables were tested for normality using the Kolmogorov–Smirnov test. Variables conforming to a normal distribution were expressed as Mean ± Standard Deviation (M ± SD), while non-normally distributed data were presented as median and interquartile range (M [Q1, Q3]). For continuous variables with normal distribution and homogeneity of variance, independent samples t-tests (for two groups) or one-way analysis of variance (ANOVA, for multiple groups) were applied. Bivariate correlations were assessed using Pearson’s correlation coefficient for normally distributed data and Spearman’s rank correlation coefficient for non-normal data.

Our data analysis employed Hierarchical Linear Modeling (HLM) because a substantial portion (68.3%) of the differences in healthcare professionals’ attitudes and behaviors were found to be attributable to the specific hospitals in which they worked. Failing to account for this “cluster effect” would be equivalent to combining employees from different hospitals, potentially leading to inaccurate conclusions. By utilizing the HLM model, we were able to more accurately capture the relationships between variables, providing a more reliable foundation for developing hospital-level management policies. In presenting the results, we primarily focused on unstandardized regression coefficients (B values). This approach was chosen due to the practical applicability of the findings. B values can be directly interpreted as the number of units by which the outcome variable (e.g., moral courage) changes for every one-unit change in a predictor variable (e.g., empathy). This interpretation is intuitive for managers, as it offers a clear understanding of the magnitude of psychological change needed to achieve meaningful practical effects. In contrast, the commonly used standardized coefficients (Beta values) may be underestimated in this study due to limitations in the measurement instruments, potentially leading to misleading conclusions about the relative importance of the variables.

### 3.6 Ethical considerations

The studies involving human participants were reviewed and approved by the Ethics Committee of Inner Mongolia (Approval No. 202504004L). All nurses who participated in this study were adults aged 18 or above. Prior to data collection, each participant provided written informed consent, confirming their voluntary participation and understanding of the study’s purpose, procedures, and potential risks. Since all participants were adults, there was no need for legal guardians or next of kin involvement in the consent process.

## 4 Results

### 4.1 Descriptive and univariate analysis

A total of 1,273 nurses participated in the study, ranging in age from 21 to 59 years, with a mean age of 34.81 years (SD = 7.12). Of these, 371 (29.1%) were internal medicine nurses, 272 (21.4%) were surgical nurses, 152 (11.9%) were obstetrics and gynecology nurses, 55 (4.3%) were pediatric nurses, 184 (14.5%) were employed in emergency, critical care, and intensive care unit, and 239 (18.8%) were from other departments. The study’s findings indicated that factors such as gender, department, education level, professional title, job position, night shifts per month, total years of work experience, self-perceived health status, and frequency of off-site training per year significantly influenced clinical nurses’ job crafting. As demonstrated in Table 1, all observed differences were statistically significant (*p* < 0.05).

**Table 1:** Single factor analysis.

	*n* (%)	Job crafting (score, Mean ± SD)	*t/F*	*p*
Gender				
Male	65 (5.1%)	86.48 ± 18.26	3.543	0.001
Female	1,208 (94.9%)	78.27 ± 16.91		
Department				
Internal medicine	371 (29.1%)	76.75 ± 16.85	3.307	0.006
Surgery	272 (21.4%)	80.25 ± 17.64		
Obstetrics and gynecology	152 (11.9%)	80.52 ± 16.93		
Pediatrics	55 (4.3%)	82.49 ± 15.54		
Emergency, critical care, and intensive care unit	184 (14.5%)	76.21 ± 16.51		
Other departments	239 (18.8%)	79.79 ± 17.21		
Ethnicity				
Han Chinese	1,037 (81.5%)	78.86 ± 16.92	1.404	0.240
Mongolian	182 (14.3%)	78.19 ± 16.56		
Hui Chinese	18 (1.4%)	83.33 ± 17.59		
Other ethnic groups	36 (2.8%)	74.08 ± 22.65		
Education level				
Associate degree or below	143 (11.2%)	82.65 ± 18.83	7.019	0.001
Bachelor’s degree	1,119 (87.9%)	78.07 ± 16.78		
Master’s degree or above	11 (0.9%)	89.91 ± 12.19		
Marital status				
Unmarried	299 (23.5%)	80.07 ± 19.00	2.569	0.077
Married without children	164 (12.9%)	80.21 ± 16.58		
Married with children	810 (63.6%)	77.87 ± 16.38		
Professional title				
Primary	545 (42.8%)	80.58 ± 18.53	8.086	<0.001
Intermediate-level	499 (39.2%)	76.38 ± 16.66		
Senior-level	229 (18.0%)	79.21 ± 13.42		
Job position				
Nurse	1,183 (92.9%)	78.42 ± 17.27	−2.482	0.015
Head nurse	90 (7.1%)	82.23 ± 13.78		
Night shifts per month				
≤4 times	479 (37.6%)	80.29 ± 15.94	3.639	0.027
5 ~ 9 times	665 (52.2%)	77.54 ± 17.26		
>9 times	129 (10.1%)	78.65 ± 19.62		
Total years of work experience				
<3 years	174 (13.7%)	82.75 ± 17.73	5.575	0.001
3 ~ 5 years	119 (9.3%)	81.39 ± 17.99		
6 ~ 10 years	291 (22.9%)	77.75 ± 18.63		
>10 years	689 (54.1%)	77.59 ± 15.85		
Employment status				
Non-established position	942 (74.0%)	78.97 ± 17.36	1.003	0.316
Established position	331 (26.0%)	77.88 ± 16.22		
Self-perceived health status				
Excellent	525 (41.2%)	86.26 ± 17.16	74.444	<0.001
Very good	349 (27.4%)	77.53 ± 14.22		
Good	327 (25.7%)	71.43 ± 13.64		
Fair	64 (5.0%)	64.23 ± 14.63		
Poor	8 (0.6%)	44.5 ± 14.76		
Frequency of off-site training per year				
1 times	1,145 (89.9%)	78.29 ± 17.10	3.522	0.015
2 times	68 (5.3%)	85.16 ± 12.56		
3 times	15 (1.2%)	77.73 ± 23.58		
4 times or more	45 (3.5%)	79.33 ± 18.28		

*Post hoc* multiple comparisons revealed that nurses with an associate degree or below exhibited significantly higher levels of job crafting compared to those with a bachelor’s degree; job crafting was significantly higher among nurses with primary and senior titles compared to those with intermediate titles; job crafting was significantly more prevalent among nurses who worked ≤4 night shifts per month compared to those working 5 ~ 9 shifts per month; nurses with <3 years of professional experience exhibited higher levels of job crafting than those with 6 ~ 10 years or >10 years of experience; nurses who perceived themselves as having better health reported higher levels of job crafting; the frequency of training also played a role; nurses who participated in training twice a year demonstrated significantly higher job crafting than those who attended training once a year, refer to Supplementary file S2.

### 4.2 Analysis of the current status of job crafting among nurses

As indicated in Table 2, the extent of job crafting among clinical nurses was moderate. A one-sample *t*-test analysis revealed that the mean scores for job crafting, career calling, regulatory focus, psychological capital, and the five personality traits were significantly above the theoretical median. These findings suggest that the nursing cohort possesses a more favorable self-assessment regarding their occupational psychological resources, such as psychological capital and regulatory focus, as well as their personality traits.

**Table 2:** Analysis of the current situation.

	Mean ± SD	Skewness coefficient	Kurtosis coefficient	Test statistic	*t*-value
Task crafting	3.51 ± 0.97	−0.18	−0.27	3.00	18.884^***^
Cognitive crafting	3.85 ± 0.84	−0.41	0.23	3.00	35.972^***^
Relational crafting	3.88 ± 0.81	−0.44	0.57	3.00	38.931^***^
Job crafting	3.75 ± 0.81	−0.21	0.21	3.00	32.785^***^
Career calling	3.83 ± 0.85	−0.53	0.43	3.00	34.823^***^
Promotion focus	4.14 ± 0.71	−1.02	2.74	3.00	57.418^***^
Prevention focus	4.03 ± 0.73	−0.85	1.99	3.00	50.225^***^
Regulatory focus	4.09 ± 0.71	−0.95	2.56	3.00	55.030^***^
Self-efficacy	4.86 ± 0.95	−1.00	1.77	3.50	51.368^***^
Hope	4.83 ± 0.95	−0.92	1.53	3.50	50.141^***^
Resilience	4.85 ± 0.94	−0.93	1.60	3.50	51.121^***^
Optimism	4.81 ± 1.05	−1.05	1.31	3.50	44.609^***^
Psychological capital	4.84 ± 0.93	−0.91	1.55	3.50	51.456^***^
Extraversion	5.07 ± 1.19	−0.03	−0.16	4.00	32.287^***^
Agreeableness	4.52 ± 1.28	0.52	−0.21	4.00	14.467^***^
Conscientiousness	4.75 ± 1.17	0.41	0.03	4.00	22.697^***^
Emotional stability	4.82 ± 1.18	0.26	−0.02	4.00	24.791^***^
Openness	5.04 ± 1.12	0.04	0.32	4.00	33.207^***^
TIPI-C	4.84 ± 1.04	0.51	0.68	4.00	28.745^***^

*** represents *p* < 0.001. On the Likert 5 scale, a score of 3 represents neutrality, so the test value is 3; on the Likert 6 scale, a score of 3.5 represents neutrality, so the test value is 3.5; and on the Likert 7 scale, a score of 4 represents neutrality, so the test value is 4.

### 4.3 Pearson correlation analysis

The Pearson correlation analysis results indicated that career calling, regulatory focus, psychological capital, and ten-item personality inventory in China (TIPI-C) were all positively correlated with job crafting (*r* = 0.797, *p* < 0.01; *r* = 0.766, *p* < 0.01; *r* = 0.772, *p* < 0.01; *r* = 0.539, *p* < 0.01), as detailed in Supplementary file S3.

### 4.4 Common method bias test

To assess common method variance, we employed the unmeasured latent method factor approach recommended by Podsakoff et al. (43). We compared the theoretical five-factor model with a model that included an additional common method factor. The theoretical five-factor model demonstrated acceptable fit indices: *χ*^2^ = 13296.709, *df* = 2,690, CFI = 0.839, TLI = 0.834, RMSEA = 0.056. The five-factor plus method factor model yielded the following fit indices: *χ*^2^ = 11135.785, *df* = 2,615, CFI = 0.871, TLI = 0.863, RMSEA = 0.051. Model comparison showed that adding the common method factor improved the CFI by 0.032. According to the criterion established by Cheung and Rensvold (44), a change in CFI (ΔCFI) greater than 0.01 indicates a substantial improvement. The ΔCFI in this study exceeded 0.01, suggesting the presence of some common method variance. However, given that the baseline model already exhibited an acceptable fit and the improvement in model fit was modest, we conclude that while common method variance is present, it does not pose a substantial threat to the core conclusions of this study.

### 4.5 Hierarchical linear model

To explore the multilevel factors influencing nurses’ job crafting, a hierarchical linear model (HLM) was established. Independent variables included those found to be statistically significant (*p* < 0.05) in the univariate analysis, as well as career calling, dimensions of the TIPI, and nurse regulatory focus. Job crafting was entered as the dependent variable. The random effects analysis indicated a significant variance component at the hospital level (variance = 107.674, Wald Z = 2.319, *p* = 0.020). The intra - class correlation coefficient (ICC), which measures the proportion of total variance in a variable that is attributable to group—level (in this case, hospital level) differences, was 0.683, suggesting that 68.3% of the total variance was attributable to differences between hospitals, thereby supporting the use of a multilevel modeling approach. The model demonstrated acceptable fit indices (AIC = 8612.447; BIC = 8622.696). Results from the fixed effects analysis revealed that career calling, extraversion, conscientiousness, nurse regulatory focus, department, education level, years of work experience, and self-perceived health status were significant predictors of nurses’ job crafting. Specifically, nurses working in pediatrics reported significantly higher levels of job crafting than those in other departments. Nurses holding a master’s degree or above exhibited significantly greater job crafting than those with a bachelor’s degree or an associate degree and below. Moreover, nurses who perceived their health status as “poor” reported significantly lower levels of job crafting compared with those who rated their health as “fair” or “good” In contrast, nurses with less than 3 years and 3 ~ 5 years of work experience demonstrated significantly higher levels of job crafting compared with those who had more than 10 years of experience (Table 3).

**Table 3:** Hierarchical linear model.

		Unstandardized coefficients	SE	*t*-value	*p*-value	95% CI
Fixed effects						
(Constant)		30.83	5.00	6.17	0.00	[20.89, 40.77]
Department	Internal medicine	−0.19	0.62	−0.31	0.76	[−1.40, 1.02]
	Surgery	0.29	0.66	0.44	0.66	[−1.00, 1.57]
	Obstetrics gynecology	0.63	0.77	0.82	0.42	[−0.89, 2.15]
	Pediatrics	2.79	1.08	2.58	0.01	[0.67, 4.91]
	Emergency, critical care and intensive care unit	−0.44	0.73	−0.60	0.55	[−1.88, 1.00]
	Other	0b	0.00			
Education level	Associate degree or above	−6.07	2.31	−2.63	0.01	[−10.59, −1.55]
	Bachelor’s degree	−5.47	2.22	−2.47	0.01	[−9.83, −1.12]
	Master’s degree or above	0b	0.00			
Total years of experience	<3 years	1.72	0.75	2.29	0.02	[0.24,3.19]
	3 ~ 5 years	2.25	0.82	2.74	0.01	[0.64,3.87]
	6 ~ 10 years	1.07	0.55	1.95	0.05	[−0.01, 2.14]
	>10 years	0b	0.00			
Self-perceived health status	Excellent	4.94	2.64	1.87	0.06	[−0.24, 10.12]
	Very good	4.08	2.63	1.55	0.12	[−1.08, 9.24]
	Good	5.18	2.61	1.99	0.05	[0.06, 10.30]
	Fair	6.86	2.71	2.53	0.01	[1.54, 12.19]
	Poor	0b	0.00			
Career calling		0.36	0.04	8.45	0.00	[0.28, 0.44]
Nurse regulatory focus		0.545	0.05	11.945	0.00	[0.46, 0.64]
Extraversion		0.381	0.15	2.629	0.01	[0.10, 0.67]
Conscientiousness		−0.699	0.16	−4.404	0.00	[−1.01, −0.39]
Random effects						
Intercept (between-hospitals)		107.67	46.43	2.32	0.020	[46.24, 250.71]
Residual (within-hospital)		49.87	2.01	24.81	<0.001	[46.08, 53.97]

(1) This table includes only statistically significant variables. Job crafting was used as the dependent variable. The two dimensions of Nurse Regulatory Focus (promotion focus and prevention focus) and Psychological Capital (along with its dimensions) exhibited substantial multicollinearity. To ensure the accuracy of the linear regression results, these variables were excluded from the analysis as independent variables for job crafting. (2) Model Summary: Estimation Method = Restricted Maximum Likelihood (REML); Model Fit Indices: −2 Restricted Log Likelihood = 8608.45, Akaike’s Information Criterion (AIC) = 8612.45, Schwarz’s Bayesian Criterion (BIC) = 8622.70; Intraclass Correlation Coefficient (ICC) = 0.684. (3) Explanation of Variance: Based on a comparison with the null model, the marginal R^2^ (the proportion of variance explained by the fixed effects alone) for the full model is 0.462, and the conditional R^2^ (the proportion of variance explained by both the fixed and random effects) is 0.580. This indicates that the model has good explanatory power. However, caution should be exercised when interpreting effect sizes related to the TIPI-C personality traits. Due to its two-item structure, the TIPI-C may result in restricted variance and reduced reliability; consequently, any standardized coefficients or variance-based effect sizes derived from it may be underestimated or distorted. Therefore, the primary interpretation in this study is based on the unstandardized coefficients (B).

### 4.6 Mediating effect analysis

**Figure 1:**
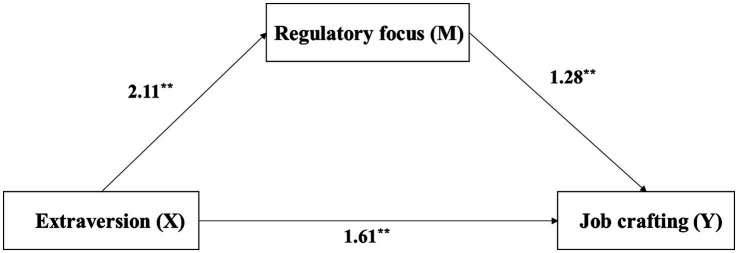
The mediating role of regulatory focus in the relationship between extraversion and job crafting. Path values are unstandardized coefficients (B). ***p < 0.001.

**Figure 2:**
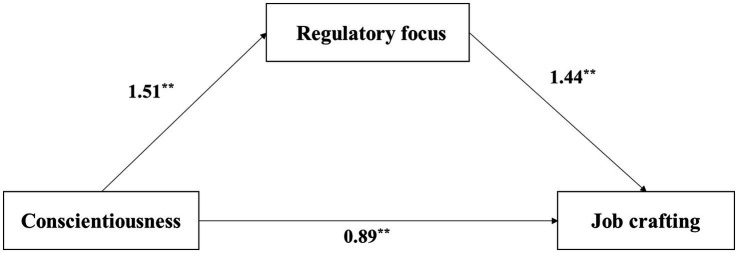
The mediating role of regulatory focus in the relationship between conscientiousness and job crafting. Path values are unstandardized coefficients (B). ***p < 0.001.

To examine the mediating role of regulatory focus between personality traits and job crafting, this study employed Hayes’ (2018) PROCESS macro (Model 4) with 5,000 bootstrap samples. The path analysis results (see Figures 1, 2; Table 4) revealed the following: First, for extraversion, its total effect on job crafting was significant (*B* = 4.31, *p* < 0.001). Extraversion significantly and positively predicted regulatory focus (*B* = 2.11, *p* < 0.001), which in turn significantly and positively predicted job crafting (*B* = 1.28, *p* < 0.001). The indirect effect through regulatory focus was 2.69, with a 95% bootstrap confidence interval [2.40, 2.99] that did not include zero, indicating a statistically significant mediation effect. After including the mediator, the direct effect of extraversion on job crafting remained significant (*B* = 1.61, *p* < 0.001), confirming partial mediation. The overall regression model, including extraversion and regulatory focus, was significant (*R*^2^ = 0.62, *F* = 1032.80, *p* < 0.001). For conscientiousness, its total effect on job crafting was also significant (*B* = 3.07, *p* < 0.001). Conscientiousness significantly and positively predicted regulatory focus (*B* = 1.51, *p* < 0.001), and regulatory focus significantly predicted job crafting (*B* = 1.44, *p* < 0.001). The indirect effect via regulatory focus was 2.18, with a 95% bootstrap confidence interval [1.88, 2.49] that did not include zero, indicating a significant mediation effect. The direct effect of conscientiousness on job crafting remained significant after accounting for the mediator (*B* = 0.89, *p* < 0.001). This overall model was also significant (*R*^2^ = 0.60, *F* = 948.75, *p* < 0.001). In summary, regulatory focus served as a partial mediator in the relationships between both extraversion and conscientiousness, and job crafting. The proportions of the indirect effects to the total effects were 62.56% for extraversion and 70.89% for conscientiousness, indicating that the influence of these personality traits on job crafting is primarily mediated by regulatory focus, with the mediating effect playing a dominant role.

**Table 4:** Results of the mediation effect analysis and bootstrap test (*n* = 1,273).

Model	Effect type	Path	Unstandardized coefficient	BootSE	95% CI	Effect proportion (%)
Model 1	Direct effect	Extraversion → Job crafting	1.61	0.15	[1.31, 1.92]	37.44
	Indirect effect	Extraversion → Regulatory focus → Job crafting	2.69	0.15	[2.40, 2.99]	62.56
	Total effect	Extraversion → Job crafting	4.31	0.16	[3.99, 4.62]	–
Model 2	Direct effect	Conscientiousness → Job crafting	0.89	0.14	[0.62, 1.17]	29.11
	Indirect effect	Conscientiousness → Regulatory focus → Job crafting	2.18	0.16	[1.88, 2.49]	70.89
	Total effect	Conscientiousness → Job crafting	3.07	0.19	[2.71, 3.44]	–

## 5 Discussion

### 5.1 Analysis of the current status of nurses’ job crafting and group differences

This study found that the extent of job crafting among clinical nurses was moderately high, consistent with Park’s findings (45). Younger nurses were more likely to engage in task crafting to meet professional demands, while older nurses focused more on relational crafting. This aligns with Zhang’s research on age stratification among nursing staff (46). Additionally, nurses with less than 3 years of experience showed significantly higher levels of task crafting compared to other groups. This supports Benner’s theory of novice development, which suggests that new nurses develop clinical competence by actively adapting their work styles during occupational socialization (47). Conversely, job crafting levels declined among nurses with 6–10 years of experience, indicating an increased risk of burnout at this career stage and highlighting the need to address the “mid-career trap.” Furthermore, the study observed a U-shaped distribution in relation to professional titles. Nurses with primary titles exhibited greater motivation for job crafting, driven by career exploration needs. Those with senior titles were more involved in clinical teaching and systemic change. In contrast, nurses with intermediate titles faced role overload due to demands in developing their specialties, leading to decreased motivation for job crafting (48–50).

Nurses working low-frequency night shifts (≤4 times per month) exhibited significantly higher levels of job crafting compared to those on high-frequency shifts (5–9 times per month). This suggests that frequent disruptions to circadian rhythms may hinder proactive behaviors, as they deplete psychological resources. This finding is consistent with Van’s research on the cognitive load experienced by shift-based nursing staff (51, 52). Additionally, the impact of continuing education appeared nonlinear, with nurses in the biannual out-of-home training cohort showing superior performance. Zhuang’s study supports this by showing that training frequency beyond a certain threshold can lead to learning fatigue, reducing the effectiveness of knowledge translation (53). Overall, these findings indicate that job crafting among a young, highly mobile nursing workforce results from the dynamic interaction between individual characteristics and the organizational environment. Future research should explore the moderating effects of contextual factors, such as department type, to refine strategies for career development interventions.

### 5.2 Analysis of factors influencing nurses’ job crafting

In line with the Job Demands-Resources (JD-R) model, an occupational stress and well - being framework that posits two core categories of job characteristics—job demands and job resources, as proposed by Shan et al. (54), the findings of this study suggest that nurses’ job crafting is systematically influenced by both environmental resources (department) and individual resources (including education, health perception, personality traits, Career Calling, and Nurse Regulatory Focus). At the environmental level, surgical nurses significantly enhance their work efficacy through task integration, supporting Jakobsson et al.’s (55) conclusion regarding adaptive strategies in high-stress departments. However, the incidence of innovative behavior among pediatric nurses was significantly higher than conventional predictions, which contrasts with Zelesniack et al.’s (56) observation of the inhibitory effect of Conscientiousness on innovation. It is hypothesized that the unique demands of pediatric patient care may mitigate the constraints imposed by professional norms. For example, multi-task coordination training could be emphasized in surgical departments, empathy communication workshops could be implemented in obstetrics and gynecology, and error-tolerant mechanisms could be introduced in pediatrics to foster innovative practices.

At the individual level, this study reveals the structural differentiation mechanism through which education level and work experience influence nurses’ job crafting. The results indicate a U-shaped distribution of job crafting behavior across nursing education levels: nurses with a master’s degree or higher exhibit stronger job crafting tendencies, leveraging their professional expertise to pursue higher career achievements (57). In contrast, nurses with an associate degree or below primarily engage in practical job crafting, driven by accumulated experience and responses to workplace challenges (58). Nurses with a bachelor’s degree are positioned at the trough of this U-shaped distribution, which is closely linked to career development bottlenecks, insufficient resource support, and an underdeveloped certification system (59). Furthermore, the study finds that career satisfaction and work environment significantly moderate nurses’ job crafting behaviors. Nurses with a bachelor’s degree in unsupportive work environments are at greater risk of burnout (60). Work experience also distinctly influences job crafting: nurses with fewer than 3 years of experience, typically early-career professionals, often exhibit high enthusiasm. Those with 3–5 years of experience have mastered routine workflows and begin pursuing personal career development (61). In contrast, nurses with over 10 years of experience, despite high job stability, frequently experience burnout and fatigue, leading to diminished job crafting and reduced self-crafting capacity (62). These measures would help revitalize their professional engagement and provide more adequate support and developmental opportunities.

At the individual level, career calling significantly and positively predicts nurses’ job crafting, indicating that nurses with a strong sense of career calling are more likely to proactively adjust their job content and roles. This finding is consistent with Esteves’ research (63). In exploring the mechanisms underlying this relationship, Solms et al. (64) found that nurses build psychological resilience through goal-oriented behaviors, such as proactive resource acquisition, which help mitigate occupational stress. The present study suggests that nurses with a strong career calling may engage in job crafting through a similar mechanism. Empirical evidence shows a robust link between career calling and job satisfaction, organizational commitment, and career development (65). Additionally, a sense of professional mission can enhance career success by strengthening psychological resilience (66). In nursing management, fostering nurses’ sense of professional mission and psychological resilience is essential for improving career satisfaction and reducing turnover intentions (67). Therefore, nursing managers should prioritize offering career development opportunities and support to enhance nurses’ sense of mission and resilience, ultimately improving care quality and nurses’ ability to transform their work.

At the individual level, regulatory focus is a significant and positive predictor of nurses’ job crafting, suggesting that nurses’ personal regulatory tendencies influence how they redefine their roles. This finding is consistent with Roczniewska’s study (68). Given the dynamic nature of the nursing profession and the diverse needs of patients (69, 70), regulatory focus indicates that nurses with a promotion focus are more likely to seek professional growth by exploring new responsibilities. In contrast, nurses with a prevention focus tend to mitigate risks by optimizing existing processes, both contributing to job crafting (71). This may reflect the nursing profession’s culture, which emphasizes achievement-oriented behaviors. Based on these findings, nursing managers can enhance nurses’ regulatory focus through tailored strategies—providing innovative task platforms for promotion focus nurses and establishing risk-warning support systems for prevention focus nurses—thereby optimizing job crafting behaviors. Future research should also differentiate between regulatory focus dimensions to offer more precise guidance for decision-making in nursing human resource management.

### 5.3 Analysis of the mediating effects of nurse job crafting

This study presents an interesting finding regarding the mediating role of regulatory focus in the relationship between nurses’ personality traits and job crafting. The results suggest that extraversion and conscientiousness significantly influence nurses’ job crafting behaviors primarily through the mediation of regulatory focus, with contribution rates of 62.56 and 70.89%, respectively. This finding is consistent with the results reported by Wu et al. (72). For extraverted nurses, when expanding their social support networks, the focus of their behavior tends to be on building emotional connections rather than pursuing instrumental goals. This aligns with the nature of extraversion, which emphasizes social interaction and emotional expression. In contrast, conscientious nurses, when engaging in quality improvement practices, often achieve a strong connection between nursing safety goals and their professional mission (73, 74). Previous research has also identified cognitive and behavioral correlates of these personality traits. For example, individuals with higher levels of extraversion have been found to perform better in change detection tasks (75), hinting at a potential link between extraversion and enhanced attentional control. They are more likely to identify opportunities for job crafting and engage in behaviors that optimize their work experience. Similarly, conscientiousness has been shown to increase work engagement, especially under high work pressure or overtime conditions (76). Nurses exhibiting high conscientiousness are more likely to perceive their work as meaningful, which promotes positive job crafting outcomes. This facilitating role of conscientiousness has also been confirmed in other studies, particularly when a sense of meaning in the work environment is heightened (77).

According to Regulatory Focus Theory, individuals with a promotion focus pursue growth, innovation, and development, are more willing to embrace challenges and take risks, and are particularly sensitive to information about progress and achievement. In contrast, individuals with a prevention focus prioritize safety and caution to avoid mistakes, contributing to stability and reliability in their work (78). Within the JD-R theoretical framework, regulatory focus serves as a core mediating variable through which personality traits influence job crafting. A promotion focus enhances opportunity identification by directing individuals toward success-oriented goals, while a prevention focus optimizes risk decision-making by directing individuals to avoid potential risks.

In conclusion, this study’s investigation into the distinct mediating pathways of regulatory focus across different personality traits has informed the development of a tailored, personality-based approach to guide head nurses in implementing effective job crafting strategies. For nurses exhibiting high levels of extraversion, whose job crafting behaviors are primarily driven by promotion focus, as documented in multiple nursing studies (31), head nurses are encouraged to leverage their social vitality and growth orientation by engaging them in meaningful roles. These include appointing them as mentors or preceptors for new staff, thereby formalizing their natural inclination toward social interaction; involving them in interdisciplinary projects and patient support groups to harness their interpersonal strengths; and offering opportunities to represent their department on hospital committees to fulfill their aspirations for visibility and career progression.

In contrast, for nurses characterized by strong conscientiousness whose job crafting is closely associated with prevention focus, as supported by research emphasizing the importance of person-job fit for well-being among nursing staff (79), managers should draw on their intrinsic sense of responsibility and attention to detail. Targeted strategies may include authorizing these nurses to develop or optimize clinical procedures and safety checklists, engaging them in quality assurance and auditing activities, and assigning them to oversee critical equipment or supply inventories. These roles not only allow their meticulousness and reliability to shine but also align their personal values with institutional priorities for patient safety and quality improvement. By adopting a personalized intervention strategy, nursing managers can move beyond the traditional one-size-fits-all model and activate the intrinsic motivational mechanisms unique to each personality profile. Such targeted approaches are likely to enhance the relevance and sustainability of job crafting efforts, leading to improved nursing performance, greater professional fulfillment, and ultimately, better patient care outcomes.

## 6 Limitations

This study has several limitations. First, the cross-sectional design and reliance on self-reported data prevent causal inferences and may introduce common method bias. These limitations restrict the practical utility of our model in informing intervention strategies, as we cannot definitively determine whether personality traits directly influence moral courage or if other unmeasured factors are at play. Although procedural and statistical remedies, such as Harman’s single-factor test, were employed to address potential bias, it cannot be completely ruled out. Future research should adopt longitudinal or experimental designs to establish temporal precedence and causality, which are crucial for developing effective training programs.

Second, the sample was drawn exclusively from tertiary hospitals in Inner Mongolia, which may limit the generalizability of the findings to other healthcare settings or cultural contexts. As a result, the relationships identified in this study may not directly apply to primary care settings or regions with different healthcare systems and cultural values, potentially leading to misapplied practices if generalized indiscriminately. Future studies should aim to include more diverse populations from various hospital levels and cultural backgrounds to test the boundary conditions of our findings and build a more universally applicable theory.

Third, regarding theoretical contributions, the use of the Ten-Item Personality Inventory (TIPI-C) presents notable conceptual limitations. While the TIPI-C is suitable for rapid screening, its two-item-per-dimension structure limits the variance and reliability of the measurements, as evidenced by the modest inter-item correlations (*r* = 0.07–0.20). These psychometric limitations directly affect the testing of our proposed mediation model. Specifically, measurement errors in both the predictor (personality) and the mediator are likely to attenuate the observed path coefficients. From a practical standpoint, this suggests that the true influence of personality on moral courage is likely stronger than our results indicate. Organizations utilizing these findings for personnel selection or development should be aware that the true effect of personality may be underestimated. Although our analysis reveals statistically significant patterns and we report unstandardized coefficients to enhance interpretability, this study is better suited for providing descriptive insights rather than offering strong confirmatory evidence for the underlying psychological mechanisms. To improve personality assessment in future research, we recommend using more comprehensive, validated multi-item instruments, such as the NEO-PI-R, for more reliable and nuanced measurements of personality traits.

It is also important to note that no sensitivity analyses or statistical corrections for measurement error were conducted in this study. Future research could benefit from methods such as structural equation modeling with latent variables to better account for measurement error and improve the accuracy of path coefficient estimates.

Finally, the proposed mediation model did not account for contextual factors such as organizational support or leadership. This narrow focus limits the model’s practical value for designing organizational interventions, as it overlooks modifiable environmental factors that could be targeted to enhance moral courage more effectively than stable personality traits. Future research should replicate these associations using more comprehensive personality instruments (e.g., NEO-PI-R) and develop multilevel models that integrate both individual- and organizational-level variables. This approach would provide a more robust test of the mechanisms involved and offer a more actionable framework for healthcare administrators.

## 7 Conclusion

Research indicates that the overall level of job crafting among clinical nurses is moderately high, influenced by both individual and environmental factors. At the individual level, factors such as education, years of experience, self-perceived health status, personality traits, career calling, and regulatory focus significantly predict job crafting behaviors. At the environmental level, nurses working in surgical, obstetrics/gynecology, and pediatric departments exhibit significantly higher job crafting capabilities compared to those in other units. Notably, personality traits such as extraversion and conscientiousness are strongly associated with job crafting, and further analysis confirms that regulatory focus plays a key mediating role in this relationship. Based on these findings, it is recommended that nursing management optimize person-job fit by systematically assessing the alignment between nurses’ personality traits and job requirements. Additionally, fostering cross-departmental exchanges of best practices could enhance adaptive job crafting skills, particularly in high-pressure environments. Finally, implementing dynamic health monitoring mechanisms can facilitate the synergistic management of psychological resources and job demands. Therefore, nursing management should adopt differentiated strategies tailored to individual personality traits. For nurses high in extraversion, assigning roles such as mentors or leaders of patient education initiatives can effectively leverage their social energy and drive for personal growth. In contrast, for nurses with strong conscientiousness, empowering them to participate in the development of safety protocols, lead quality assurance audits, or manage critical clinical equipment allows their meticulousness and focus on safety to align with organizational priorities. In addition, managers should proactively shape the work environment to facilitate adaptive job crafting. This includes implementing promotion-focused interventions to stimulate growth-oriented job crafting behaviors and establishing prevention-focused mechanisms to support stability-oriented job crafting. By aligning environmental cues with individual motivational tendencies, nursing managers can foster sustained engagement and enhance overall workforce performance.

## Data Availability

Raw data supporting the conclusions are available from the corresponding author upon reasonable request.

## Footnotes

1
https://www.wjx.cn/

## References

[1] Tims M Bakker AB Derks D. Development and validation of the job crafting scale. J Vocat Behav. (2012) 80:173–86. doi: 10.1016/j.jvb.2011.05.009

[2] Berg JM Dutton JE Wrzesniewski A. Job crafting and meaningful work (2013). doi: 10.1037/14183-005

[3] Dutton WJE. Crafting a job: revisioning employees as active crafters of their work. Acad Manag Rev. (2001) 26:179–201. doi: 10.2307/259118

[4] International Labour Organization. Decent work country programme for China 2023–2025. Beijing: International Labour Organization (2023).

[5] Tims M Bakker AB. Job crafting: towards a new model of individual job redesign. SA J Ind Psychol. (2010) 36:1–9. doi: 10.4102/sajip.v36i2.841

[6] Hu H Wang C Lan Y Wu X. Nurses' turnover intention, hope and career identity: the mediating role of job satisfaction. BMC Nurs. (2022) 21:43. doi: 10.1186/s12912-022-00821-5, 35144604 PMC8830989

[7] Zhang X Zhang C Gou J Lee SY. The influence of psychosocial work environment, personal perceived health and job crafting on nurses' well-being: a cross-sectional survey study. BMC Nurs. (2024) 23:373. doi: 10.1186/s12912-024-02041-5, 38831334 PMC11145890

[8] Harbridge R Ivanitskaya L Spreitzer G Boscart V. Job crafting in registered nurses working in public health: a qualitative study. Appl Nurs Res. (2022) 64:151556. doi: 10.1016/j.apnr.2021.151556, 35307127

[9] Han S. Nurses' job crafting, work engagement, and well-being: a path analysis. BMC Nurs. (2023) 22:405. doi: 10.1186/s12912-023-01573-6, 37904210 PMC10614409

[10] Fu H Du Y Wang Z. Authentic leadership and taking charge behavior: a moderated mediation model of psychological capital and occupational calling. Int J Environ Res Public Health. (2022) 19:5492. doi: 10.3390/ijerph19095492, 35564886 PMC9100871

[11] Cruz-Cobo C. The influence of job crafting on nurses' intent to stay: a cross-sectional study. Nurs Rep. (2024) 14:3436–44. doi: 10.3390/nursrep1404024 39585140 PMC11587447

[12] Bakker AB Demerouti E. The job demands-resources model: state of the art. J Manag Psychol. (2007) 22:309–28. doi: 10.1108/02683940710733115

[13] Wang Y Yang Q Wang L Zhang Q Li Y. The factors of job crafting in emergency nurses: regression models versus qualitative comparative analysis. BMC Nurs. (2024) 23:369. doi: 10.1186/s12912-024-02035-3, 38825685 PMC11145844

[14] Courson S Breen K Smith J Flynn A Evans J Moffitt R . Impact of COVID-19 on the level of work engagement of nurses at a small, rural hospital. J Contin Educ Nurs. (2022) 53:157–64. doi: 10.3928/00220124-20220311-05, 35357998

[15] Lee HF Chang YJ. The effects of work satisfaction and work flexibility on burnout in nurses. J Nurs Res. (2022) 30:e240. doi: 10.1097/jnr.0000000000000522, 36166364

[16] Chu X Zhang L Li M. Nurses' strengths use and turnover intention: the roles of job crafting and self-efficacy. J Adv Nurs. (2022) 78:2075–84. doi: 10.1111/jan.15124, 34859903

[17] Demerouti E Bakker AB Nachreiner F Schaufeli WB. The job demands-resources model of burnout. J Appl Psychol. (2001) 86:499–512. doi: 10.1037/0021-9010.86.3.499, 11419809

[18] Bakker AB. Job crafting among health care professionals: the role of work engagement. J Nurs Manag. (2018) 26:321–31. doi: 10.1111/jonm.12551, 28990295

[19] Galanis P Moisoglou I Papathanasiou IV Malliarou M Katsiroumpa A Vraka I . Association between organizational support and turnover intention in nurses: a systematic review and meta-analysis. Healthcare. (2024) 12:291. doi: 10.3390/healthcare12030291, 38338176 PMC10855592

[20] Gori A Arcioni A Topino E Palazzeschi L Di Fabio A. Constructing well-being in organizations: first empirical results on job crafting, personality traits, and insight. Int J Environ Res Public Health. (2021) 18:18. doi: 10.3390/ijerph18126661, 34205683 PMC8296412

[21] Wilmot MP Ones DS. A century of research on conscientiousness at work. Proc Natl Acad Sci USA. (2019) 116:23004–10. doi: 10.1073/pnas.1908430116, 31666330 PMC6859351

[22] Shdaifat E Shudayfat T Alshowkan A. The relationship between personality traits and happiness: the mediating role of emotional regulation. BMC Nurs. (2024) 23:327. doi: 10.1186/s12912-024-01959-0, 38745308 PMC11092155

[23] Semeijn JH van der Heijden B Beuckelaer AD. Personality traits and types in relation to career success: an empirical comparison using the big five. Appl Psychol. (2020) 69:538–56. doi: 10.1111/apps.12174, 41293653

[24] Jiang H Mei Y Wang X Zhao Z Lin B Wang W . Professional calling among nursing students: a latent profile analysis. BMC Nurs. (2023) 22:299. doi: 10.1186/s12912-023-01470-y, 37660012 PMC10474663

[25] Raatikainen R. Nursing care as a calling. J Adv Nurs. (1997) 25:1111–5. doi: 10.1046/j.1365-2648.1997.19970251111.x, 9181405

[26] Chen J Wang L Tang N. Half the sky: the moderating role of cultural collectivism in job turnover among Chinese female workers. J Bus Ethics. (2016) 133:487–98. doi: 10.1007/s10551-014-2395-1

[27] Kim HK Lee TK Kong WY. The interplay between framing and regulatory focus in processing narratives about HPV vaccination in Singapore. Health Commun. (2020) 35:222–32. doi: 10.1080/10410236.2018.1553022, 30526080

[28] Molero Jurado MDM Pérez-Fuentes MDC Barragán Martín AB Gázquez Linares JJ Oropesa Ruiz NF Simón Márquez MDM. Emotional intelligence components as predictors of engagement in nursing professionals by sex. Healthcare. (2020) 8:42. doi: 10.3390/healthcare8010042, 32098453 PMC7151152

[29] Molero Jurado MDM Pérez-Fuentes MDC Oropesa Ruiz NF Simón Márquez MDM Gázquez Linares JJ. Self-efficacy and emotional intelligence as predictors of perceived stress in nursing professionals. Medicina (Kaunas). (2019) 55:237. doi: 10.3390/medicina55060237, 31159453 PMC6630601

[30] Martínez ÁM Jurado MMM Pérez-Fuentes MC Martín ABB Linares JJG. Personal resources and their relationship to job crafting and burnout: a challenge for male nurses as a minority group. J Men’s Health. (2022) 18:24–37. doi: 10.22514/jomh.2022.007

[31] Paustian-Underdahl SC Halbesleben JRB Carlson DS Hamadi HY. Examining regulatory focus in the acceleration and deceleration of engagement and exhaustion cycles among nurses. Health Care Manag Rev. (2023) 48:282–90. doi: 10.1097/hmr.0000000000000375, 37192154

[32] Dvorak KJ. The theoretical development and empirical testing ofthe measure of job crafting (MJC). Colorado: Colorado State University (2014).

[33] Shixiao Z. Organizational investment to enhance nurses' work engagement strategies: Taking work remodeling as an entry point. Hangzhou: Zhejiang University (2019).

[34] Dobrow SR Tosti-Kharas J. Calling: the development of a scale measure. Pers Psychol. (2011) 64:1001–49. doi: 10.1111/j.1744-6570.2011.01234.x, 41293653

[35] Pei Yujing ZS. A study on the relationship between career calling, career commitment and work attitude of knowledge-based employees. Manag Sci. (2015) 28:103–14. doi: 10.3969/j.issn.1672-0334.2015.02.010

[36] Wallace C Chen G. A multilevel integration of personality, climate, self-regulation, and performance. Pers Psychol. (2006) 59:529–57. doi: 10.1111/j.1744-6570.2006.00046.x

[37] Shuying L. A study of the effect of work self-authenticity on adaptive performance: a dual-mediation model of moderating focus. Lanzhou: Lanzhou University (2021).

[38] Luthans F Youssef-Morgan CM Avolio BJ. Psychological capital: Developing the human competitive edge. Oxford: Oxford University Press (2007).

[39] Luo Hong HZ. Reliability analysis of the psychological capital questionnaire applied to a group of nurses. Chin J Behav Med Brain Sci. (2010) 19:853–4. doi: 10.3760/cma.j.issn.1674-6554.2010.09.028

[40] Gosling SD Rentfrow PJ Swann WB. A very brief measure of the big-five personality domains. J Res Pers. (2003) 37:504–28. doi: 10.1016/S0092-6566(03)00046-1

[41] Jinde L Reliability test of the Chinese version of the 10-item big five personality inventory (TIPI-C). Chin J Health Psychol (2013) 21:5.

[42] Stankov L. Low correlations between intelligence and big five personality traits: need to broaden the domain of personality. J Intelligence. (2018) 6:6. doi: 10.3390/jintelligence6020026, 31162453 PMC6480733

[43] Podsakoff PM MacKenzie SB Lee JY Podsakoff NP. Common method biases in behavioral research: a critical review of the literature and recommended remedies. J Appl Psychol. (2003) 88:879–903. doi: 10.1037/0021-9010.88.5.879, 14516251

[44] Cheung GW Rensvold RB. Evaluating goodness-of-fit indexes for testing measurement invariance. Struct Equ Modeling. (2002) 9:233–55. doi: 10.1207/S15328007SEM0902_5

[45] Park S Ha Y. The relationship between positive psychological capital and work engagement in clinical nurses: mediation effect of job crafting. BMC Nurs. (2025) 24:117. doi: 10.1186/s12912-024-02600-w, 39901251 PMC11789409

[46] Clendon J Walker L. Being young': a qualitative study of younger nurses' experiences in the workplace. Int Nurs Rev. (2012) 59:555–61. doi: 10.1111/j.1466-7657.2012.01005.x, 23134141

[47] Yu Q Wei R Wei Y Wu X Liang T. Psychometric evaluation of the perceived perioperative competence scale-revised among the Chinese operating room nurses: a methodological research. BMC Nurs. (2022) 21:79. doi: 10.1186/s12912-022-00853-x, 35387652 PMC8988425

[48] Xu B Zhang J Hou J Ma M Gong Z Tang S. Nurses' knowledge of peripherally inserted central catheter maintenance and its influencing factors in Hunan province, China: a cross-sectional survey. BMJ Open. (2020) 10:e033804. doi: 10.1136/bmjopen-2019-033804, 32444430 PMC7247379

[49] Zhou J Chen S. Knowledge, attitudes, and practices of NICU doctors and nurses toward prevention and control of nosocomial infection with multidrug resistant organism. Front Pediatr. (2022) 10:817030. doi: 10.3389/fped.2022.817030, 35515349 PMC9062780

[50] Liu X Li C Yan X Shi B. Psychological capital has a positive correlation with humanistic care ability among nurses. Front Psychol. (2022) 13:955627. doi: 10.3389/fpsyg.2022.955627, 36186317 PMC9524352

[51] Van Nguyen T Liu HE. A cross-sectional study on sleep disturbances and associated factors among nurses. BMC Psychiatry. (2022) 22:119. doi: 10.1186/s12888-022-03748-y, 35168602 PMC8848685

[52] Zhong H Zhou L Liao S Tang J Yue L Mo M . Effects of a fixed nurse team in the orthopaedic surgery operating room on work efficiency and patient outcomes: a propensity score-matched historically controlled study. BMC Nurs. (2022) 21:248. doi: 10.1186/s12912-022-01027-5, 36068566 PMC9450373

[53] Zhuang J Mou Q Zheng T Gao F Zhong Y Lu Q . A serial mediation model of social media addiction and college students' academic engagement: the role of sleep quality and fatigue. BMC Psychiatry. (2023) 23:333. doi: 10.1186/s12888-023-04799-5, 37173670 PMC10176952

[54] Shan B Liu X Gu A Zhao R. The effect of occupational health risk perception on job satisfaction. Int J Environ Res Public Health. (2022) 19:19. doi: 10.3390/ijerph19042111, 35206297 PMC8872356

[55] Jakobsson J Jangland E Engström M Malmström M Drott J. Work conditions influencing professional development of specialist nurses in surgical care explored using the job demand-resources theory: a qualitative study. J Adv Nurs. (2023) 79:2610–21. doi: 10.1111/jan.15618, 36843299

[56] Zelesniack E Oubaid V Harendza S. Defining competence profiles of different medical specialties with the requirement-tracking questionnaire - a pilot study to provide a framework for medial students' choice of postgraduate training. BMC Med Educ. (2021) 21:46. doi: 10.1186/s12909-020-02479-6, 33435986 PMC7801870

[57] Sönmez B Gül D İspir Demir Ö Emiralioğlu R Erkmen T Yıldırım A. Antecedents and outcomes of nurses' subjective career success: a path analysis. J Nurs Scholarsh. (2021) 53:604–14. doi: 10.1111/jnu.12660, 33829661

[58] Jansson J Josse Eklund A Larsson M Nilsson J. Prehospital care nurses' self reported competence: a cross-sectional study. Int Emerg Nurs. (2020) 52:100896. doi: 10.1016/j.ienj.2020.100896, 32763799

[59] Chang S Han K Cho Y. Association of Happiness and Nursing Work Environments with job crafting among hospital nurses in South Korea. Int J Environ Res Public Health. (2020) 17:17. doi: 10.3390/ijerph17114042, 32517109 PMC7312226

[60] Carolan C Davies CL Crookes P McGhee S Roxburgh M. COVID 19: disruptive impacts and transformative opportunities in undergraduate nurse education. Nurse Educ Pract. (2020) 46:102807. doi: 10.1016/j.nepr.2020.102807, 32502757 PMC7255089

[61] Duffield C Baldwin R Roche M Wise S. Job enrichment: creating meaningful career development opportunities for nurses. J Nurs Manag. (2014) 22:697–706. doi: 10.1111/jonm.12049, 23463905

[62] Rudman A Arborelius L Dahlgren A Finnes A Gustavsson P. Consequences of early career nurse burnout: a prospective long-term follow-up on cognitive functions, depressive symptoms, and insomnia. EClinicalMedicine. (2020) 27:100565. doi: 10.1016/j.eclinm.2020.100565, 33150328 PMC7599295

[63] Esteves T Lopes MP. Crafting a calling: the mediating role of calling between challenging job demands and turnover intention. J Career Dev. (2017) 44:34–48. doi: 10.1177/0894845316633789

[64] Solms L van Vianen AEM Theeboom T Koen J de Pagter APJ de Hoog M. Keep the fire burning: a survey study on the role of personal resources for work engagement and burnout in medical residents and specialists in the Netherlands. BMJ Open. (2019) 9:e031053. doi: 10.1136/bmjopen-2019-031053, 31694848 PMC6858141

[65] Ada HM Dehom S D'Errico E Boyd K Taylor EJ. Sanctification of work and hospital nurse employment outcomes: an observational study. J Nurs Manag. (2021) 29:442–50. doi: 10.1111/jonm.13162, 32961596 PMC8247287

[66] Zhang L Liang X Cheng N Han L Jia Y Wang R . Psychological resilience mediates sense of professional mission and career success in Chinese intensive care unit nurses: a cross-sectional study. BMC Nurs. (2024) 23:607. doi: 10.1186/s12912-024-02271-7, 39218871 PMC11367826

[67] Trybou J Malfait S Gemmel P Clays E. Nursing staff and their team: impact on intention to leave. Int Nurs Rev. (2015) 62:489–96. doi: 10.1111/inr.12216, 26390899

[68] Roczniewska M Bakker AB. Burnout and self-regulation failure: a diary study of self-undermining and job crafting among nurses. J Adv Nurs. (2021) 77:3424–35. doi: 10.1111/jan.14872, 33955050

[69] He F He RX. Systematic nursing interventions in gastric cancer: a randomized controlled study. World J Clin Cases. (2022) 10:1843–51. doi: 10.12998/wjcc.v10.i6.1843, 35317163 PMC8891775

[70] Afshar M Sadeghi-Gandomani H Masoudi Alavi N. A study on improving nursing clinical competencies in a surgical department: a participatory action research. Nurs Open. (2020) 7:1052–9. doi: 10.1002/nop2.485, 32587724 PMC7308675

[71] Liu D Zhang Y Geng X. The effect of chronic regulatory focus and social comparison on undergraduates' intertemporal choices under gain-loss frame. Front Psychol. (2022) 13:1076304. doi: 10.3389/fpsyg.2022.1076304, 36687826 PMC9853201

[72] Wu ZF Wang S. Status quo and influencing factors of team job crafting among clinical nurses. J Nurs (China). (2025) 32:71–5. doi: 10.16460/j.issn1008-9969.2025.05.071

[73] Hsu MC Ouyang WC. Effects of integrated moral reasoning development intervention for Management of Violence in schizophrenia: a randomized controlled trial. J Clin Med. (2022) 11:1169. doi: 10.3390/jcm11051169, 35268258 PMC8911519

[74] Huang Q Zhang K Huang Y Bodla AA Zou X. The interactive effect of stressor appraisals and personal traits on employees' procrastination behavior: the conservation of resources perspective. Psychol Res Behav Manag. (2023) 16:781–800. doi: 10.2147/prbm.S399406, 36950311 PMC10025370

[75] Hahn S Buttaccio DR Hahn J Lee T. Personality and attention: levels of neuroticism and extraversion can predict attentional performance during a change detection task. Q J Exp Psychol (Hove). (2015) 68:1041–8. doi: 10.1080/17470218.2015.1032986, 25801545

[76] Hagen T De Caluwé E Bogaerts S. Personality moderators of the cross-sectional relationship between job demands and both burnout and work engagement in judges: the boosting effects of conscientiousness and introversion. Int J Law Psychiatry. (2023) 89:101902. doi: 10.1016/j.ijlp.2023.101902, 37321135

[77] Frieder RE Wang G Oh IS. Linking job-relevant personality traits, transformational leadership, and job performance via perceived meaningfulness at work: a moderated mediation model. J Appl Psychol. (2018) 103:324–33. doi: 10.1037/apl0000274, 29016164

[78] Liu CE Hu C Xie W Liu T He W. The moderated-mediation effect of workplace anxiety and regulatory focus in the relationship between work-related identity discrepancy and employee innovation. Int J Environ Res Public Health. (2020) 17:17. doi: 10.3390/ijerph17176121, 32842458 PMC7503295

[79] Ghazzawi R Bender M Daouk-Öyry L van de Vijver FJR Chasiotis A. Job crafting mediates the relation between creativity, personality, job autonomy and well-being in Lebanese nurses. J Nurs Manag. (2021) 29:2163–74. doi: 10.1111/jonm.13357, 33960053 PMC8596648

