# Supplementary material for: Influencing factors and mediating mechanisms of job crafting in clinical nursing practice
Source: Front Public Health. 2025 Dec 18;13:1711339. doi: 10.3389/fpubh.2025.1711339 (PMC12756456; doi:10.3389/fpubh.2025.1711339)
Supplement: Supplementary file 1 [file Supplementary_file_1.doc]

***Supplementary File 1***

Sampling methods:

A three-stage stratified sampling method was used in this study, which was implemented as follows:

- The initial phase, termed "alliance city sampling," involved segmenting the Inner Mongolia into three principal areas—East, Central, and West—based on its geographic and economic attributes. This division encompassed nine prefectural-level cities and three leagues. A non-equal proportional stratified sampling method was employed to select two league or prefecture-level cities from each region, taking into account factors such as sampling workload, cost, and the degree of cooperation from the sample population. Consequently, six league-municipal administrative units were included in the sample, with two from each of the eastern, central, and western regions, ensuring balanced regional representation.
- In the second phase, "hospital sampling," stratified sampling was conducted within the selected allied cities or prefecture-level cities. This was done in equal proportions, based on the regional distribution characteristics of the region's tertiary hospitals, which comprised 12 in the east, 22 in the center, and 7 in the west. The sampling quota was established based on the proportion of hospitals in each region relative to the total number of hospitals: 29.3% in the east, 53.6% in the center, and 17.1% in the west. This was determined using the principle of equal proportional distribution, resulting in the selection of 4 hospitals in the east (calculated as 12/41×12), 6 in the center (22/41×12), and 2 in the west (7/41×12), culminating in a total of 12 tertiary-level hospitals sampled.
- In the third phase, nurse sampling was conducted using a stratified whole cluster sampling method within the selected hospitals. Departmental stratification was applied according to the actual departmental configurations of the hospitals, categorizing them into internal medicine, surgery, gynecology and pediatrics, emergency departments, and other specialties. Subsequently, whole cluster sampling was employed, whereby 2 to 4 complete nursing units were randomly selected as sampling clusters from each departmental stratum in each hospital. All on-duty registered nurses within these clusters were ultimately included in the sample.
