# Supplementary material for: Influencing factors and mediating mechanisms of job crafting in clinical nursing practice
Source: Front Public Health. 2025 Dec 18;13:1711339. doi: 10.3389/fpubh.2025.1711339 (PMC12756456; doi:10.3389/fpubh.2025.1711339)
Supplement: Supplementary file 2 [file Supplementary_file_2.docx]

***Supplementary File 2***

Descriptive and univariate analysis

1 The results of Bonferroni correction showed that job crafting was significantly higher for associate degree or below nurses than for bachelor's degree nurses (Table 1, Figure 1)

Table 1 Bonferroni correction

| I | J | Mean difference (I-J) | SE | P | 95% confidence interval | |
| --- | --- | --- | --- | --- | --- | --- |
|  |  |  |  |  | Lower limit | Upper limit |
| Associate degree or below | Bachelor's degree | 4.57796* | 1.509 | 0.007 | 0.9603 | 8.1957 |


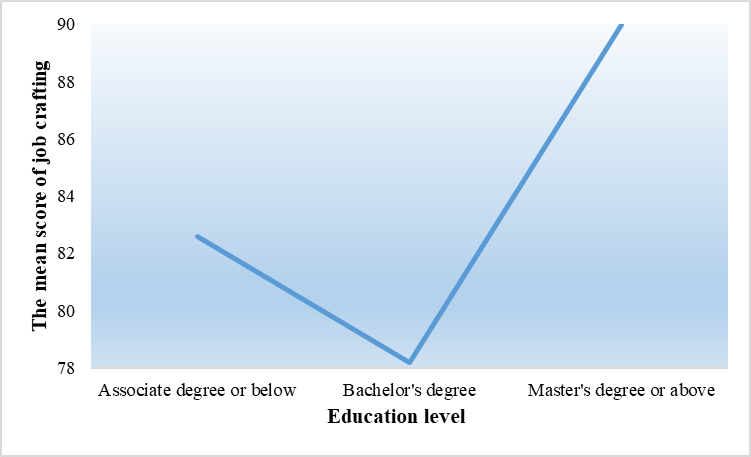


Figure 1

2 The results of Tamhane T2 test showed that job crafting was significantly higher for primary and senior title nurses than for intermediate title nurses (Table 2, Figure 2)

Table 2 Tamhane T2 test

| I | J | Mean difference (I-J) | SE | P | 95% confidence interval | |
| --- | --- | --- | --- | --- | --- | --- |
|  |  |  |  |  | Lower limit | Upper limit |
| Primary-level | Intermediate-level | 4.19688* | 1.08924 | 0.000 | 1.5919 | 6.8019 |
| Intermediate-level | Senior-level | -2.82047* | 1.15884 | 0.045 | -5.5961 | -0.0449 |


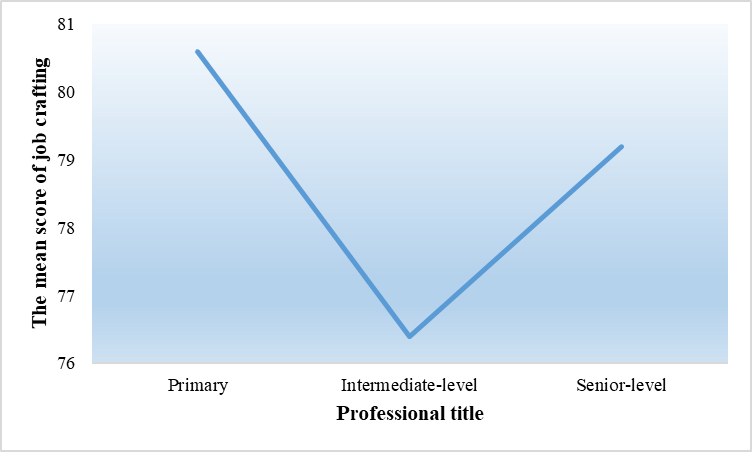


Figure 2

3 The results of Tamhane T2 test showed that job crafting was significantly higher for nurses with ≤4 night shifts per month than for nurses with 5-9 times (Table 3, Figure 3)

Table 3 Tamhane T2 test

| I | J | Mean difference (I-J) | SE | P | 95% confidence interval | |
| --- | --- | --- | --- | --- | --- | --- |
|  |  |  |  |  | Lower limit | Upper limit |
| ≤4 times | 5-9 times | 2.75451* | 0.98937 | 0.016 | 0.3885 | 5.1206 |


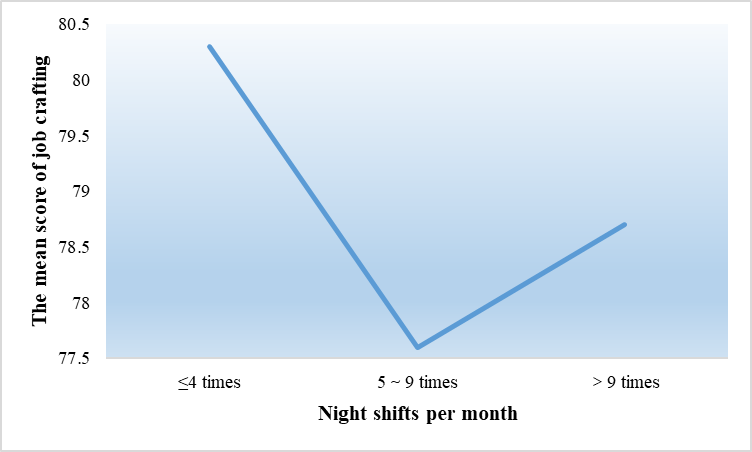


Figure 3

4 The results of Tamhane T2 test showed that job crafting was significantly higher for nurses with <3 years of work experience than for nurses with 6-10 years and >10 years (Table 4, Figure 4)

Table 4 Tamhane T2 test

| I | J | Mean difference (I-J) | SE | P | 95% confidence interval | |
| --- | --- | --- | --- | --- | --- | --- |
|  |  |  |  |  | Lower limit | Upper limit |
| ＜3years | 6-10 years | 5.00030* | 1.73167 | 0.024 | 0.4203 | 9.5803 |
| ＜3 years | ＞10 years | 5.16071* | 1.47315 | 0.003 | 1.2535 | 9.0679 |


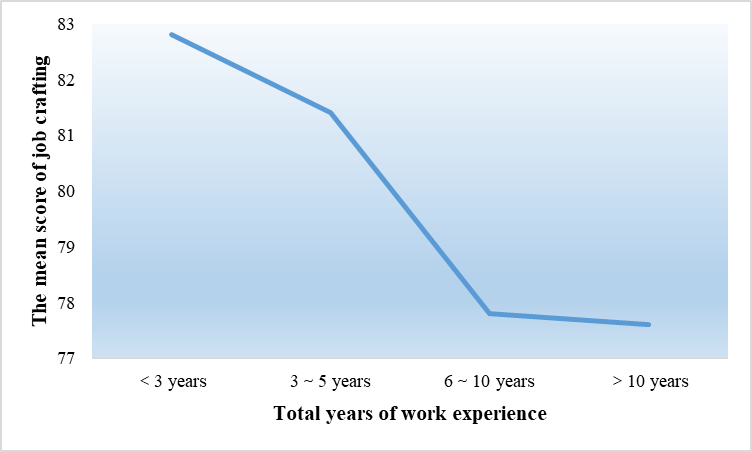


Figure 4

5 The results of Tamhane T2 test showed that nurses with better self-perceived health have higher job crafting (Table 5, Figure 5)

Table 5 Tamhane T2 test

| I | J | Mean difference (I-J) | SE | P | 95% confidence interval | |
| --- | --- | --- | --- | --- | --- | --- |
|  |  |  |  |  | Lower limit | Upper limit |
| Excellent | Very good | 8.73564* | 1.06771 | 0.000 | 5.7385 | 11.7328 |
| Excellent | Good | 14.82861* | 1.06289 | 0.000 | 11.8447 | 17.8125 |
| Excellent | Fair | 22.02848* | 1.97626 | 0.000 | 16.3494 | 27.7075 |
| Excellent | Poor | 41.76286* | 5.27361 | 0.001 | 20.9461 | 62.5796 |
| Very good | Excellent | -8.73564* | 1.06771 | 0.000 | -11.7328 | -5.7385 |
| Very good | Good | 6.09297* | 1.07133 | 0.000 | 3.0838 | 9.1021 |
| Very good | Fair | 13.29285* | 1.98081 | 0.000 | 7.6018 | 18.9839 |
| Very good | Poor | 33.02722* | 5.27532 | 0.004 | 12.2141 | 53.8404 |
| Good | Excellent | -14.82861* | 1.06289 | 0.000 | -17.8125 | -11.8447 |
| Good | Very good | -6.09297* | 1.07133 | 0.000 | -9.1021 | -3.0838 |
| Good | Fair | 7.19988* | 1.97822 | 0.005 | 1.5155 | 12.8843 |
| Good | Poor | 26.93425* | 5.27435 | 0.012 | 6.1190 | 47.7495 |
| Fair | Excellent | -22.02848* | 1.97626 | 0.000 | -27.7075 | -16.3494 |
| Fair | Very good | -13.29285* | 1.98081 | 0.000 | -18.9839 | -7.6018 |
| Fair | Good | -7.19988* | 1.97822 | 0.005 | -12.8843 | -1.5155 |
| Fair | Poor | 19.73438 | 5.53124 | 0.061 | -0.7245 | 40.1932 |
| Poor | Excellent | -41.76286* | 5.27361 | 0.001 | -62.5796 | -20.9461 |
| Poor | Very good | -33.02722* | 5.27532 | 0.004 | -53.8404 | -12.2141 |
| Poor | Good | -26.93425* | 5.27435 | 0.012 | -47.7495 | -6.1190 |
| Poor | Fair | -19.73438 | 5.53124 | 0.061 | -40.1932 | 0.7245 |


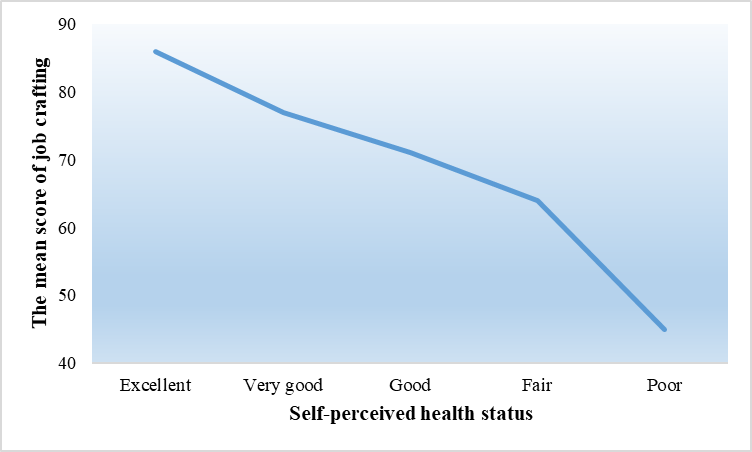


Figure 5

6 The results of Tamhane T2 test showed that job crafting was significantly higher for nurses who trained 2 times a year than for nurses who trained 1 time (Table 6, Figure 6)

Table 6 Tamhane T2 test

| I | J | Mean difference (I-J) | SE | P | 95% confidence interval | |
| --- | --- | --- | --- | --- | --- | --- |
|  |  |  |  |  | Lower limit | Upper limit |
| 1 time | 2 times | -6.87006* | 1.60493 | 0.000 | -11.1964 | -2.5437 |


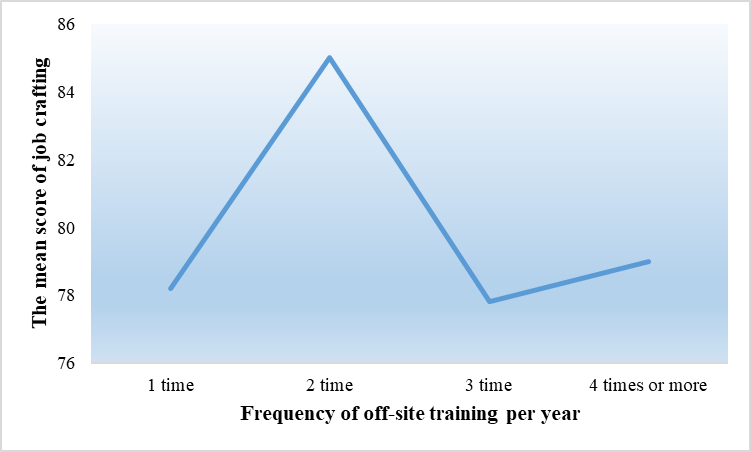


Figure 6
