# Supplementary material for: Influencing factors and mediating mechanisms of job crafting in clinical nursing practice
Source: Front Public Health. 2025 Dec 18;13:1711339. doi: 10.3389/fpubh.2025.1711339 (PMC12756456; doi:10.3389/fpubh.2025.1711339)
Supplement: Supplementary file 3 [file Supplementary_file_3.docx]

***Supplementary File 3***

Table 1 Pearson correlation analysis

|  | 1 | 2 | 3 | 4 | 5 | 6 | 7 | 8 | 9 | 10 | 11 | 12 | 13 | 14 | 15 | 16 | 17 | 18 | 19 |
| --- | --- | --- | --- | --- | --- | --- | --- | --- | --- | --- | --- | --- | --- | --- | --- | --- | --- | --- | --- |
| 1 Task Crafting | 1 |  |  |  |  |  |  |  |  |  |  |  |  |  |  |  |  |  |  |
| 2 Cognitive Crafting | 0.780^**^ | 1 |  |  |  |  |  |  |  |  |  |  |  |  |  |  |  |  |  |
| 3 Relationship Crafting | 0.754^**^ | 0.882^**^ | 1 |  |  |  |  |  |  |  |  |  |  |  |  |  |  |  |  |
| 4 Promotion Focus | 0.570^**^ | 0.723^**^ | 0.736^**^ | 1 |  |  |  |  |  |  |  |  |  |  |  |  |  |  |  |
| 5 Prevention Focus | 0.638^**^ | 0.774^**^ | 0.772^**^ | 0.909^**^ | 1 |  |  |  |  |  |  |  |  |  |  |  |  |  |  |
| 6 Self-Efficacy | 0.661^**^ | 0.749^**^ | 0.749^**^ | 0.798^**^ | 0.839^**^ | 1 |  |  |  |  |  |  |  |  |  |  |  |  |  |
| 7 Hope | 0.651^**^ | 0.716^**^ | 0.732^**^ | 0.783^**^ | 0.810^**^ | 0.920^**^ | 1 |  |  |  |  |  |  |  |  |  |  |  |  |
| 8 Resilience | 0.640^**^ | 0.724^**^ | 0.723^**^ | 0.791^**^ | 0.822^**^ | 0.918^**^ | 0.941^**^ | 1 |  |  |  |  |  |  |  |  |  |  |  |
| 9 Optimism | 0.632^**^ | 0.702^**^ | 0.678^**^ | 0.739^**^ | 0.789^**^ | 0.880^**^ | 0.912^**^ | 0.907^**^ | 1 |  |  |  |  |  |  |  |  |  |  |
| 10 Extraversion | 0.544^**^ | 0.568^**^ | 0.561^**^ | 0.548^**^ | 0.605^**^ | 0.668^**^ | 0.687^**^ | 0.672^**^ | 0.657^**^ | 1 |  |  |  |  |  |  |  |  |  |
| 11 Agreeableness | 0.459^**^ | 0.417^**^ | 0.429^**^ | 0.377^**^ | 0.428^**^ | 0.505^**^ | 0.516^**^ | 0.508^**^ | 0.498^**^ | 0.693^**^ | 1 |  |  |  |  |  |  |  |  |
| 12 Conscientiousness | 0.411^**^ | 0.383^**^ | 0.384^**^ | 0.392^**^ | 0.427^**^ | 0.474^**^ | 0.482^**^ | 0.482^**^ | 0.458^**^ | 0.675^**^ | 0.762^**^ | 1 |  |  |  |  |  |  |  |
| 13 Emotional Stability | 0.364^**^ | 0.369^**^ | 0.372^**^ | 0.354^**^ | 0.386^**^ | 0.424^**^ | 0.426^**^ | 0.440^**^ | 0.386^**^ | 0.631^**^ | 0.787^**^ | 0.749^**^ | 1 |  |  |  |  |  |  |
| 14 Openness | 0.425^**^ | 0.455^**^ | 0.468^**^ | 0.476^**^ | 0.503^**^ | 0.543^**^ | 0.564^**^ | 0.568^**^ | 0.544^**^ | 0.679^**^ | 0.700^**^ | 0.755^**^ | 0.689^**^ | 1 |  |  |  |  |  |
| 15 Job crafting | 0.916^**^ | 0.947^**^ | 0.935^**^ | 0.719^**^ | 0.776^**^ | 0.769^**^ | 0.748^**^ | 0.744^**^ | 0.718^**^ | 0.598^**^ | 0.468^**^ | 0.422^**^ | 0.395^**^ | 0.481^**^ | 1 |  |  |  |  |
| 16 Career Calling | 0.697^**^ | 0.777^**^ | 0.760^**^ | 0.783^**^ | 0.865^**^ | 0.830^**^ | 0.833^**^ | 0.820^**^ | 0.831^**^ | 0.647^**^ | 0.480^**^ | 0.461^**^ | 0.391^**^ | 0.510^**^ | 0.797^**^ | 1 |  |  |  |
| 17 Nurse regulatory focus | 0.619^**^ | 0.767^**^ | 0.772^**^ | 0.976^**^ | 0.978^**^ | 0.839^**^ | 0.816^**^ | 0.826^**^ | 0.782^**^ | 0.591^**^ | 0.412^**^ | 0.419^**^ | 0.379^**^ | 0.501^**^ | 0.766^**^ | 0.844^**^ | 1 |  |  |
| 18 Psychological capital | 0.669^**^ | 0.749^**^ | 0.750^**^ | 0.808^**^ | 0.845^**^ | 0.967^**^ | 0.978^**^ | 0.974^**^ | 0.946^**^ | 0.695^**^ | 0.524^**^ | 0.491^**^ | 0.436^**^ | 0.574^**^ | 0.772^**^ | 0.856^**^ | 0.846^**^ | 1 |  |
| 19 TIPI-C | 0.503^**^ | 0.499^**^ | 0.504^**^ | 0.488^**^ | 0.534^**^ | 0.595^**^ | 0.609^**^ | 0.608^**^ | 0.579^**^ | 0.838^**^ | 0.903^**^ | 0.898^**^ | 0.879^**^ | 0.867^**^ | 0.539^**^ | 0.567^**^ | 0.523^**^ | 0.619^**^ | 1 |

Note: *represents *p* < 0.05, **represents *p* < 0.01.
